# Palliative Care Professionals’ Message to Others: An Ethnographic Approach

**DOI:** 10.3390/ijerph18105348

**Published:** 2021-05-17

**Authors:** Carla Reigada, Carlos Centeno, Edna Gonçalves, Maria Arantzamendi

**Affiliations:** 1 ATLANTES Research Group, Institute for Culture and Society, University of Navarra, 31009 Pamplona, Spain; ccenteno@unav.es (C.C.); marantz@unav.es (M.A.); 2 Health Research Institute of Navarra (IdiSNA), 31009 Pamplona, Spain; 3 Palliative Medicine Department, Clínica Universidad de Navarra, 31009 Pamplona, Spain; 4 Palliative Care Service, Centro Hospitalar Universitário de São João, E.P.E., 4200-319 Porto, Portugal; ednafgoncalves@gmail.com

**Keywords:** palliative care, message, communication, interprofessional

## Abstract

Introduction: Palliative care continues to be misunderstood within the world of healthcare. Palliative care professionals are key agents for promoting a greater understanding of their field. This study aims to examine the messages, both implicit and explicit, that palliative care professionals transmit about themselves and their work within their teams and to other health professionals. Methods: Focused ethnographic secondary analysis, exploring the interactions of palliative care professionals as it happens at everyday work. An inductive thematic analysis was developed from 242 h of observation of the daily work practices of palliative care professionals, focusing on their interactions with others. The data was coded without predefined categories, and the analysis was performed independently by two researchers. Results: Palliative professionals communicate that they are part of an active team working in an organized manner. They value and feel proud of their work. Despite the intensity of their work, these professionals are always available to others, to whom they demonstrate a clear professional identity. They convey their expertise in alleviating suffering, respectful behavior and collaborative ability. Conclusion: Professionals, in their daily work, communicate through their messages the essence of palliative care. It is essential that palliative care professionals perceive themselves as potential influencers and explicitly transmit the reasons for their intervention. Otherwise, others will perpetuate the myths, misunderstandings, and lack of a positive reputation for palliative care.

## 1. Introduction

Communication plays a central role in palliative care and is considered an essential competency for palliative specialists, ensuring good relationship with patients, families and healthcare professionals [1]. Palliative care professionals perceive that their communication and the intention of their interventions are clear and of great value to others [2]. However, messages transmitted by palliative care professionals within their own team and to other health professionals, are frequently considered divergent and frequently inaccurate [3].

It is known that palliative care professionals’ messages to patients and families are focused on their well-being and marked by an attitude of availability [4], and that is recognized by the patients themselves [5]. However, despite the fact that palliative care professionals are communication experts [1], society continues to associate “death” with palliative care [6,7]. Even within the healthcare field, many colleagues are reluctant to collaborate with palliative care professionals and understand palliative care to be a second-rate discipline [8,9].

Although we can find scientific evidence regarding patient–carers–team intercommunication, there is a lack of studies on the effective communication about palliative care in healthcare teams. Healthcare professionals understand palliative care as an ambiguous and distorted subject. Quite often, the role of the specialist in palliative medicine is unknown and unspecified [9].

Transmitting a clear, positive universal message about palliative care is a complex task that requires an innovative strategy [10]. As palliative professionals are key agents in communicating to others what palliative care is, this study aims to understand the messages, both implicit and explicit, that palliative care professionals transmit about themselves within their team and to other health professionals.

## 2. Materials and Methods

### 2.1. Study Design, Recruitment

Secondary analysis of a focused ethnographic study to understand what palliative care professionals transmit about palliative care and how it is perceived among themselves and patients and carers [4]. The focused ethnographic is a methodology that allows a deep understanding of a particular problem and context, in this case palliative care, exploring cultural aspects, values and beliefs of a specific group of people [11,12].

Data were collected from February to September 2018 through 242 h of continuous participant observation and informal conversations, by the same researcher (C.R., expert in PC and ethnography), in three palliative care teams from different regions of Spain (physicians = 10, nurses = 6, psychologists = 3, social workers = 1).

To select the teams observed we used data from the Spanish Association of Palliative Care (SECPAL) which identified a total of 24 complete teams (physicians, nurses, psychologists and social workers) providing palliative care in Spain, in hospitals (consultation within an acute hospital), home care (home care), inpatient care (PC beds in acute hospitals) and external clinical care (external consultation).

Inclusion criteria included teams with at least 80% of their professionals trained in PC and consenting to the researcher’s observation of daily routine for at least 20 h per week. Once the inclusion criteria were considered, three services from different types of regimes were selected: one service from the public system; one mixed service from the private and public system; one service from the private system.

All teams have more than eight years of experience in PC. The teams linked to the public system appeared mainly through primary care, using the knowledge of “personalized medicine” that the primary care teams provide. Later, the in-hospital care in both public and private systems, was recognized of major impact on community care. All PC teams, more at the private level, bet on training (courses, discussion of cases) of other professionals as a way to reach collaboration.

### 2.2. Access

First the directors of each PC team were contacted by email asking for permission to conduct the study which was presented to the respective team members. Written consent was obtained from the directors and professionals of each team. Since the study included a participant observation, the consent of patients, families, and other health professionals was obtained by verbal, on a continuous and interactive approach.

### 2.3. Data Collection

To facilitate the integration of the researcher (C.R.) into each team, a “contact person” was appointed per team whose function was to facilitate the relationship. Each observation period began in the morning with the team meeting. The researcher followed the team during the day observing and participating in the care activity. Field notes were recorded out of sight of the team using a cell phone, and later recorded in more detail in a Microsoft Word document to avoid memory bias. Informal conversations were held with the professionals asking directly about a given situation and directly recording their perspective. A reflective journal was used so that the researcher could write down his or her interpretations (which were not observations) separately, which allowed for reflection on the observation process and the building of analytical concepts. Finally, discussions were held with the research team and researchers together at the end of each observation period and also internal documents of each PC service team were consulted.

### 2.4. Analysis

The content analysis was developed inductively, coded with no predefined categories and conducted independently by two researchers (C.R. and M.A.), considering the following research question: “What is the message about palliative care that these professionals transmit to other health professionals in their daily practice?” This helped to focus the analysis on the interaction and communication between the elements of different teams rather than on specific clinical interventions of care or specialization.

The analysis began with conversations between researchers, at the same time as the data collection. The field notes were coded independently by two researchers (C.R. and M.A.) who discussed the coding that each had assigned, paying attention to the differences. In the case of variations, they held further discussions to review their field notes and discuss the comments, behaviors, and attitudes of each described interaction. Later, the researchers began to classify the codes into categories and possible themes.

The findings were presented to two other researchers (E.G. and C.C.), and together they discussed the final coding, paying attention to the differences.

### 2.5. Ethics

This study was approved by the Ethics Committee of the University of Navarra (No. 2018.009). This document facilitated the approval of the three institutions involved in the study. All participants provided their consent for the study.

## 3. Results

By observing the daily interactions of palliative care professionals, we perceived that within the palliative care team (a) they form active, organized teams; (b) they value and feel proud of their work; (c) they are always available to others despite intense work schedules. In their interactions with other professionals, we observed that a) they demonstrate clear professional identity; (b) they are experts in alleviating suffering; (c) they work respectfully and collaboratively with everyone.

Please find the Palliative care professionals’ key messages in the Supplementary Materials Figure S1.

### 3.1. Messages Observed in Interactions within the Palliative Care Team

#### 3.1.1. They Form Active and Organized Teams

The palliative care professionals observed in this study hold team meetings every day at a designated time and place. They interact with one another in an open and interdisciplinary way, and they review each clinical situation systematically. They transmit being an active group, working together in an organized manner.

From a researcher’s diary: *‘… (the palliative care team) … They start the day with a team meeting. A round table allows them to look at each other and discuss patients. The meeting starts when everyone is prepared.’*

They use the same terms, although they sometimes assign nuance to them—for example, to the word ‘caregiver’.

From a discussion between a researcher and a physician: *‘The physician does not have the same perception as the nurse. For the nurse, the “caregiver” is the main person who takes care of the patient, while the physician says, “I do not agree. I understand the caregiver as family.”’*

#### 3.1.2. They Value the Impact Their Work Makes on Patients’ Lives

Palliative care professionals’ value and feel proud of their work. It is typical for them to receive letters of gratitude, notes and offerings, which they proudly share with each other—for instance, by displaying them on a wall, or by storing them in a folder accessible to all team members. This emotional environment is valued and seems to have a role in their wellbeing as it is openly displayed as a shared recognition of their joint effort. It is a symbolic language where the messages of patients and families are a reward for their work.

An example from a researcher’s diary states: *‘There is a medical office next to the nursing area. It’s an office full of pictures and “thank you” notes all over the walls. These are messages from patients and families.’*

#### 3.1.3. They Are Always Available to Patients and Families, Despite Their Intense Work Schedule

During work hours, when palliative care team members are not with patients, they allow themselves only short breaks and move quickly between clinical encounters, revealing an urgent and efficient effort to achieve all of their tasks on time. They are always running. Their interaction as a team revealed “hard work and lack of time” to do everything that should be done. However, in spite of this intense problem-solving schedule, they are always available to serve patients and caregivers.

Observation fieldnote: *‘The professionals walk at a fast pace through the hospital corridors. They are determined to solve problems. They have little time to stop, drink coffee, eat, or give way to the self-care they recommend to others.’ A palliative care team member says that they ‘are professionals with a small stomach and large bladder!’*

Whenever the team meets a new patient or family member, their collective attitude shifts radically. No longer in a hurry, they focus all of their attention on the person they are going to serve and instead of fast-paced work, they transmit calm and tranquility.

Observation fieldnote: *‘The team [previously walking fast in the hospital corridor] sees a woman sitting with her son at the hospital window. The team sits on the bed, touches the patient and talks about the family. [Laughs.] They spent more than 30 min sitting with this lady who told them her life experiences.’*

### 3.2. Messages Transmitted by Palliative Care Professionals in Daily Clinical Interactions with Other Healthcare Professionals.

#### 3.2.1. They Display an Unequivocal Professional Identity to Others

When palliative care professionals introduce themselves to other teams, they clearly identify their area of expertise using expressions such as ‘we are from palliative care’ or ‘we are the palliative care team’. This contrasts with their typical procedure for introducing themselves to families, in which they seem to initially avoid saying the name of their field (‘palliative care’). With colleagues, however, they state their professional identity without hesitation, with a persistent and positive attitude.

Direct observation: *‘Today, I focus my observation on the team’s physician. He answers the telephone mentioning his name and the name of the service: “Hello, I’m (name), from the palliative care service…” During the conversation, he mentions again the name of the service and spells it out.’*

#### 3.2.2. They Are Experts in Relieving Suffering from Severe Illness

Palliative care professionals demonstrate their availability to others as well as their specialized skills, when handling complex situations such as the end-of-life decision-making process. In fact, when faced with difficult situations, specialized teams (e.g., oncology, neurology) often call upon the palliative care team, recognizing their capacity to help. Palliative care professionals use their communication and emotional support skills in these complex cases to facilitate discussion between specialists, encouraging intercollaboration, respect and proximity.

From a researcher’s diary: *‘The intensive care unit called the palliative care professionals to help in the decision-making process of a complex situation. The palliative care team immediately answered to this call. They found a case of a previously autonomous, healthy older woman who suffered severe head trauma following a fall. She has advance directives stating that she does not want invasive interventions. Given that this is an acute situation, it is necessary to decide on performing or not invasive procedures to the patient. The palliative care team encouraged the conversation between different specialists.’*

#### 3.2.3. They Collaborate with and Respect Their Colleagues

Palliative care professionals maintain an open and positive attitude towards colleagues of all specialties. They demonstrate a desire to collaborate with others and actively seek their contributions. They prefer direct contact—in person or by phone—to indirect forms of communication, such as formal requests or messages sent from the clinical information system.

From a discussion between a researcher and physician: *‘The palliative care physician says, “Our task is to involve the patient’s family doctor. It is mandatory, it is our job. We don’t want to replace them; we want to add a complementary service.”’*

From a discussion between a researcher and physician, in which the researcher asks, *‘How is the patient referred to you in this service?’: ‘Directly! If the patient is admitted at the hospital, the colleague or the attending physician can contact me directly. It has been working well.’*

## 4. Discussion

We did not find any specific study that shows how palliative care professionals transmit, directly or indirectly, the palliative care message between themselves and other professionals. This analysis illustrates the messages palliative care professionals transmit to others, in accordance with the principles and philosophy of palliative care. Cooperation is one of the values that best characterize palliative care teams, which does not seem to be readily understood by other professionals [13]. It is important for all healthcare professionals to understand that palliative care staff are eager to collaborate with them.

Palliative care professionals are aware of the positive impacts their work has on patients and their families, and they receive their words of gratitude with pride. They even share them within their teams, which facilitates the provision of care and promotes a welcoming atmosphere, as some studies show [14,15].

In this study palliative care professionals convey their expertise in relieving severe suffering in patients who are seriously ill, as well as strong communication skills and decision-making abilities. This finding was also observed in a study by Melender et al. [16] which underscored the social and problem-solving skills of palliative care physicians. The International Association for Hospice and Palliative Care (IAHPC) developed a consensus-based definition of palliative care that focuses on the relief of serious suffering. To dispel misunderstandings about their profession, palliative care workers may choose to emphasize the alleviation of suffering in the construction of their professional identity [17].

The availability of palliative care professionals has been identified as a central aspect of the message they transmit to patients and families [4]. This study finds that their constant willingness to help is also part of the message that is transmitted to other healthcare professionals about palliative care [2]. During the observation period no discussion with other health professionals was witnessed. It was identified that palliative care services tend to involve family doctors. Likewise, it was observed that family doctors or other professionals directly phoned PC professionals, if something came up. The latter could be considered an appropriate and timely access to specialist’s palliative care services, that has been reported as a factor to support good working partnership. It could be argued that there was certain collaborative approach in these services. It would be interesting to explore further how this had been promoted, as it is common to experience challenges [18].

In summary, our observations illustrate that palliative care professionals transmit messages daily about their collaborative and organizational ability, as well as their high levels of expertise. However, we also observe that these messages are transmitted implicitly rather than explicitly, which may impede understanding of the palliative care field. Palliative care professionals need to reconsider conveying “what they say they do” by transferring “why they do it.” Transmitting a message in an objective and direct way helps the receiver to perceive a congruent and real message. When they explain why a patient receives palliative care in a certain situation, we tell stories, real experiences and, in that way, we express values [19]. These values are the “gold way” to attract attention, and train others’ understanding, feeling and knowledge on palliative care. This communication strategy (speaking directly about the reason for a palliative intervention), will encourage others to repeat the same stories and, perhaps, the image about palliative care will be more authentic and coherent [20].

Myths and misunderstandings still surround palliative care [6,21]. It is crucial that professionals develop and transmit clear messages about their work if they are to seek further integration into the healthcare system [18]. Thus, we suggest that palliative care professionals adopt explicit methods of conveying their contributions to their patients, their families and the health system. Through discussion and explanation, professionals promote the social recognition that palliative care deserves.

## 5. Strengths and Limitations

This study is a secondary study, conducted in a limited time, that aims to open the topic about the message conveyed by PC professionals to other professionals. By participant observation, messages were understood by the interaction of professionals. Data were discussed systematically with other researchers, analyzed by two researchers and results discussed with the teams involved to increase credibility. The results show common messages from three different PC services in different communities in Spain. Caution is needed when considering the transferability of the results. Although it is understood that a common culture exists, there may be other factors that influence the messages of other PC professionals. It is recommended that future study further explores this research question and how other professionals perceive the message that PC professionals transmit.

## 6. Conclusions

Intercommunication skills are crucial for clinical decision making, difficult conversations about death and dying, and also for the dissemination of the real palliative care message. Palliative care professionals should recognize their role as influential speakers, revealing more explicitly the objectives of their interventions, decreasing myths, misunderstandings, and lack of positive awareness on palliative care. The palliative professionals, in their daily work, communicate through their messages the essence of palliative care. The educational attitude and the interprofessional collaboration is essential to promote the understanding of what is palliative care and palliative care professionals’ contribution.

## Data Availability

The data presented in this study are available upon request from the corresponding author. The data are not publicly available due to privacy restrictions.

## Supplementary Materials

The following are available online at https://www.mdpi.com/article/10.3390/ijerph18105348/s1, File S1: “How do I want you to know me? Palliative care professionals’ message to other professionals.”
