# Supplementary material for: Palliative Care Professionals’ Message to Others: An Ethnographic Approach
Source: Int J Environ Res Public Health. 2021 May 17;18(10):5348. doi: 10.3390/ijerph18105348 (PMC8156736; doi:10.3390/ijerph18105348)
Supplement: Supplementary file 1 [file ijerph-18-05348-s001.zip › ijerph-1204674-supplementary.pdf]

How do I want you to know me?
Palliative care professionals' message to other professionals.

Carla Reigada (1,2), Carlos Centeno (1,2, 3), Edna Gonçalves (4), Maria Arantzamendi (1,2)

(1) ATLANTES Research Group; Institute for Culture and Society; University of Navarra (Spain).
(2) Health Research Institute of Navarra (IdiSNA) (Spain).
(3) Palliative Medicine Department, Clínica Universidad de Navarra (Spain).
(4) Palliative Care Service, Centro Hospitalar Universitário de São João, E.P.E. (Portugal).

Reigada, C. *et al*. Palliative Care Professionals´ Message to Others: An Ethnographic Approach.
Int. J. Environ. Res. Public Health 2021, 18, xx.

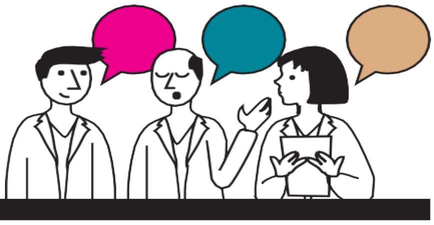

Within PC professional team

1 Active & organized team
- Daily team meetings
- The same terms, different nuances
- Discussing patients

2 They value the impact of their work
- Pictures and "thank you" notes on the walls

3 Always available for patients & families in the middle of an intense work schedule
- Fast-paced work when they not see patients
- Short breaks
- Slow-paced work when they see patients
- Personalized care: transmitting calm and tranquility

With other healthcare professionals

4 Display their professional identity
- Often saying: «we are the PC team»

5 Experts in relieving suffering from severe illness
- End-of-life decision-making process
- Facilitating discussion between specialists
- Communication skills and emotional support

6 Inter-collaboration, respecting other healthcare professionals
- Never replacing other professionals
- Face-to-face or by phone
- Interaction between different specialties
- Desire to collaborate with others

ics Universidad de Navarra
Institute for Culture and Society

ATLANTES: Human Dignity, Advanced Illness and Palliative Care Institute for Culture and Society | University of Navarra
Edificio Biblioteca de Humanidades (Entrada Sur). Campus Universitario s/n, 31009, Pamplona. Navarra (Spain)
Tel: + 34 948 42 56 00 | Ext: 802276 | @ATLANTESPC

©mgm
